# Draft Genome Sequences of Six Strains Isolated from the InSight Spacecraft and Associated Surfaces Using Oxford Nanopore- and Illumina-Based Sequencing

**DOI:** 10.1128/MRA.01161-19

**Published:** 2020-05-21

**Authors:** Daniel L. Vera, Arman Seuylemezian, Kyle S. Landry, Ryan Hendrickson

**Affiliations:** a Jet Propulsion Laboratory, California Institute of Technology, Biotechnology and Planetary Protection Group, Pasadena, California, USA; b Liberty Biosecurity, Expeditionary and Special Programs Division, Worcester, Massachusetts, USA; University of Southern California

## Abstract

Whole-genome sequencing and annotation have allowed planetary protection engineers to assess the functional capabilities of microorganisms isolated from spacecraft hardware and associated surfaces. Here, we report draft genomes of six strains isolated from the InSight mission, determined using Oxford Nanopore- and Illumina-based sequencing.

## ANNOUNCEMENT

The Interior Exploration using Seismic Investigations, Geodesy, and Heat Transport (InSight) spacecraft was launched in May 2018 to explore the interior structure of Mars using the HP_3_ and SEIS instruments. Because it was classified as a planetary protection (PP) sensitive mission, microbial cleanliness requirements were imposed on the InSight mission, and PP sampling was performed using the NASA standard spore assay throughout the life cycle of the project leading up to launch on 5 May 2018 (1). The assay selects for hardy organisms capable of surviving heat shock at 80°C for 15 min and growing on Trypticase soy agar (TSA) incubated for 72 h at 32°C. Resulting colonies are subcultured and archived for long-term storage following previously established procedures (2). Flight hardware components were sampled during final closeout activities dictated by integration timelines. Isolates generated throughout the life cycle of the project were routinely identified with matrix-assisted laser desorption ionization–time of flight mass spectrometry (MALDI-TOF MS) using previously established procedures (2). Of particular interest were six strains isolated from various spacecraft surfaces and surfaces of cleanrooms where the spacecraft was assembled. These six strains originally were identified as belonging to the same species using MALDI-TOF MS and subsequently were subjected to whole-genome sequencing and functional annotation for further characterization.

Isolates were cultured on TSA plates and incubated for 24 h; once the purity of the culture was confirmed, DNA was extracted using phenol-chloroform extraction and ethanol precipitation (3). High-molecular-weight DNA (>20 kb) was isolated using a BluePippin system (Sage Science, Beverly, MA, USA) with a High Pass Plus cassette. Nanopore libraries were generated with the SQK-LSK109 1D genomic ligation kit (Oxford Nanopore Technologies, Oxford Science Park, UK). Reads were generated with an R9.4.1 flow cell using a 48-h script. Default parameters were used for all software tools unless otherwise noted. Reads were base called with Guppy v2.3.1+1b9405b6 (4), demultiplexed with Deepbinner v0.2.0 (5), trimmed of adapters with Porechop v0.2.4, and assembled with Canu v1.8 (6). For Illumina-based polishing, libraries were sequenced on a MiSeq platform with paired-end 300-bp v3 chemistry. Reads were trimmed and filtered using Cutadapt v2.5 (7). Reads were then aligned and used to polish the assemblies with NextPolish v1.1.0 using default parameters (8). The assembled draft genomes were submitted for annotation using the NCBI Prokaryotic Genome Annotation Pipeline (PGAP). Assembly statistics for all six strains are provided in Table 1.

**TABLE 1:** Genome statistics for six draft genomes of *Bacillus megaterium* strains isolated from the InSight spacecraft and associated surfaces

Strain name	Source of isolation	Date of isolation (mo/day/yr)	GenBank accession no.	SRA accession no. for:		G+C content (%)	No. of contigs	No. of Illumina reads	*N*_50_ for genome assembly (bp)	Genome size (bp)	*N*_50_ for Oxford Nanopore reads (bp)	No. of protein-coding genes	Genome coverage (×)	16S rRNA sequence similarity to Bacillus megaterium ATCC 14581^T^ (%)
				Oxford Nanopore reads	Illumina reads									
IN_103	Secondary payload P-POD^*a*^	7/22/2014	JAAMPL000000000	SRR11356414	SRX8078840	37.59	41	1,918,356	5,074,523	6,394,712	20,315	4,793	1,378	100
IN_1371	SLC-3e launch pad floor near cleanroom tent enclosures	5/1/2018	JAAMPP000000000	SRR11307905	SRX8079841	37	57	1,912,992	5,195,541	8,206,464	23,866	5,384	485	99.93
IN_903	Atlas V payload fairing isolation diaphragm	4/4/2018	JAAMPO000000000	SRR11347569	SRX8077313	38.34	42	1,392,823	5,167,376	6,046,948	18,639	4,231	303	99.59
IN_866	SLC-3e launch pad garment change room	1/17/2018	JAAMPN000000000	SRR11342714	SRX8079423	38.94	76	1,222,128	5,132,859	6,755,230	20,416	5,901	665	99.86
IN_430	InSight spacecraft flight solar arrays	6/14/2017	JAAMPM000000000	SRR11306942	SRX8080615	37	39	1,792,819	5,070,380	7,379,828	19,838	4,300	587	99.66
IN_327	P-POD	1/17/2014	JAAEBE000000000	SRR11306541	SRX8080640	37.76	12	1,392,696	5,087,266	5,758,857	14,110	4,064	201	99.66

a P-POD, poly-picosatellite orbital deployer.

Taxonomic identification was performed using both average nucleotide identity (ANI) analysis using the Ortho-ANI algorithm (9) and pairwise comparisons of the 16S rRNA sequences extracted from whole-genome sequences against both the nonredundant/nucleotide and 16S rRNA type strain databases using BLASTn. Based on these techniques, all six strains belonged to Bacillus megaterium.

Several strains were annotated with putative genes that may have potential applications in the biotechnological or pharmaceutical industries. Strain IN_103 had a putative gene coding for a branched-chain amino acid (BCAA) aminotransferase, which catalyzes the formation of α-ketoacids and BCAAs (10). IN_903 had putative genes coding for 5-aminolevulinate synthase, which catalyzes the formation of tetrapyrroles (precursors to hemes); this enzyme has been utilized to biosynthetically produce tetrapyrrole compounds (11). Strain IN_866 had a putative gene coding for a flavin reductase, which has been utilized in gene-directed prodrug therapies to target tumor hypoxia (12).

### Data availability.

The draft genomes of all six strains have been deposited in DDBJ/EMBL/GenBank under the accession numbers provided in Table 1.
